# *Nicotiana benthamiana* Class 1 Reversibly Glycosylated Polypeptides Suppress Tobacco Mosaic Virus Infection

**DOI:** 10.3390/ijms241612843

**Published:** 2023-08-16

**Authors:** Kamila A. Kamarova, Natalia M. Ershova, Ekaterina V. Sheshukova, Eugene A. Arifulin, Natalia L. Ovsiannikova, Alexandra A. Antimonova, Andrei A. Kudriashov, Tatiana V. Komarova

**Affiliations:** 1 Vavilov Institute of General Genetics Russian Academy of Sciences, 119991 Moscow, Russia; kamila.kamarova@yandex.ru (K.A.K.); ershova@vigg.ru (N.M.E.);; 2 Belozersky Institute of Physico-Chemical Biology, Lomonosov Moscow State University, 119991 Moscow, Russia

**Keywords:** reversibly glycosylated polypeptide, plasmodesmata, intercellular transport, callose, UDP-arabinopyranose mutase, tobacco mosaic virus

## Abstract

Reversibly glycosylated polypeptides (RGPs) have been identified in many plant species and play an important role in cell wall formation, intercellular transport regulation, and plant–virus interactions. Most plants have several *RGP* genes with different expression patterns depending on the organ and developmental stage. Here, we report on four members of the RGP family in *N. benthamiana*. Based on a homology search, NbRGP1-3 and NbRGP5 were assigned to the class 1 and class 2 RGPs, respectively. We demonstrated that *NbRGP1–3* and *5* mRNA accumulation increases significantly in response to tobacco mosaic virus (TMV) infection. Moreover, all identified class 1 NbRGPs (as distinct from NbRGP5) suppress TMV intercellular transport and replication in *N. benthamiana*. Elevated expression of *NbRGP1–2* led to the stimulation of callose deposition at plasmodesmata, indicating that RGP-mediated TMV local spread could be affected via a callose-dependent mechanism. It was also demonstrated that NbRGP1 interacts with TMV movement protein (MP) in vitro and in vivo. Therefore, class 1 NbRGP1–2 play an antiviral role by impeding intercellular transport of the virus by affecting plasmodesmata callose and directly interacting with TMV MP, resulting in the reduced viral spread and replication.

## 1. Introduction

The reversibly glycosylated polypeptides (RGPs) were so named due to their ability to reversibly bind sugars in vitro by autoglycosylating a conserved arginine residue [1,2,3,4]; however, the function of these proteins in vivo remained unclear. Later, when UDP-Arap/UDP-Araf mutase activity was identified [5], the RGPs were given a new name—UDP-Arap mutases (UAMs). Surprisingly, not all proteins in the family possess UAM activity. Therefore, based on this feature, they were divided into two classes: catalytically active RGPs were classified as class 1 RGPs, whereas the others, which lacked UAM activity, were classified as class 2. A range of 1 to 5 RGPs were identified in different species, both dicots and monocots. The majority of the known RGPs were expected to have UAM activity based on sequence similarity and were included in class 1. However, experimental evidence for UAM activity has only been obtained for *Arabidopsis thaliana* RGPs 1–3 [6], *Oryza sativa* UAMs 1 and 3 [5], *Hordeum vulgare* UAMs 1–3 [7], and *Nicotiana tabacum* UAMs 1–4 [8].

RGPs have also been demonstrated to play an important role in growth and development: they act cooperatively to provide sufficient UDP-Araf for polysaccharide synthesis of the cell wall and are highly expressed in actively growing tissues [6,9,10]. Abiotic stresses such as wounding and dehydration induce *RGP* expression, as it was shown in a study of an *RGP1* promoter from *Gossypium hirsutum* in transgenic tobacco plants [11]. Chromium (Cr) stress leads to the accumulation of OsRGP3 protein in rice roots and callose in cell walls, which, together with many other factors accompanying Cr stress, provides an enhanced immobilization of Cr ions and protects rice roots against Cr toxicity [12]. In general, environmental stresses trigger a cascade of defense reactions and modulation of the intercellular transport in plant tissues via changes in the plasmodesmata (Pd) permeability [13,14]. Among multiple Pd affecting factors, RGPs are regarded as negative regulators [15,16,17,18]. 

Several studies indicate that RGPs contribute to plant–virus interactions: tobacco mosaic virus (TMV) infection spread was impeded in *AtRGP2:GFP* transgenic tobacco [15], whereas *RGP* silencing in *N. benthamiana* stimulated TMV intercellular spread [16]. *AtRGP2:GFP* overexpression in *N. tabacum* led to an increased callose deposition around plasmodesmata (Pd), which allowed the proposal of a putative mechanism for TMV intercellular movement restriction via callose-mediated decrease in Pd permeability [15]. Another member of the RGP family—UDP-glucose:protein transglucosylase (UPTG) from *Solanum lycopersicum* [19,20]—was shown to interact with tomato leaf curl virus (TLCV) V1 capsid protein; expression of SlUPTG1 in *N. benthamiana* plants in a transient TLCV replication assay increased the accumulation of viral DNA [20].

One of the first discovered members of the RGP family, salt-extractable wall-associated 41 kDa protein (SeWAP41), was isolated from the cell wall fraction of maize seedling mesocotyls. Immunogold staining revealed punctate localization of the protein in association with Pd and Golgi membranes [21]. Co-localization with the Golgi membranes was also demonstrated by Dhugga et al. [2] for *Pisum sativum* RGP. Later, numerous studies confirmed the association of RGPs with the Golgi apparatus in many different species [4,5,6,9,22]. With regard to plasmodesmal localization, however, the data are more controversial. Besides SeWAP41, Pd localization was demonstrated and confirmed by plasmolysis for SlUPTG:GFP in *Allium cepa* epidermal cells after biolistic transformation [20] and AtRGP2:GFP in transgenic tobacco plants [9,15]. However, AtRGP:YFP translational fusions gave no Pd fluorescence in transgenic *A. thaliana* plants [6,22]. This discrepancy between the observations for intracellular localization could be explained by the ectopic expression of the studied RGPs in a foreign plant as compared with the plant of origin and probably by the difference in the intracellular traffic of these proteins depending on the species. 

To understand whether *N. benthamiana* RGPs share features with the described RGPs, and reveal the role of NbRGPs in TMV infection, we used transient expression in *N. benthamiana* as an alternative to stably transformed plants. We have identified at least four members of the RGP family in the *N. benthamiana* genome and designated them as NbRGPs 1, 2, 3, and 5 based on their similarity to the known RGPs from *A. thaliana* and *N. tabacum*. Based on a homology search, NbRGP5 belongs to class 2, whereas the other three, NbRGP1–3, belong to class 1. We revealed an increased accumulation of all four *NbRGP* mRNAs in *N. benthamiana* leaves with systemic TMV infection. Moreover, class 1 NbRGP expression was demonstrated to suppress viral reproduction and local spread. To elucidate the mechanism underlying this effect, we have shown that (i) NbRGP1 and 2 stimulate Pd callose deposition and (ii) NbRGP1 directly interacts with TMV movement protein (MP) in vitro and in vivo. Based on the obtained results we hypothesize that class 1 NbRGP1 and 2 have a dual-mode action against the virus leading to the limitation of TMV local spread, having both general (via a callose-dependent mechanism) and specific (impediment to MP functioning) actions. Therefore, they play the role of antiviral cellular factors.

## 2. Results

### 2.1. NbRGP Search, Sequencing, and Expression Pattern Analysis

In previous studies, AtRGP1–4 properties and functions were studied in *Nicotiana benthamiana* plants [9], but no RGP from *N. benthamiana* was characterized. RGPs are shown to have a high degree of amino acid identity both within one species and between different species [10]. The *N. benthamiana* genome is only partially assembled and annotated, which poses additional difficulties in searching for genes with high similarity. To optimize the search, we relied on the confirmed sequences of RGP proteins from the UniProt database (https://www.uniprot.org/ (accessed on 9 July 2021)) for *A. thaliana*, *O. sativa*, *H. vulgare*, *P. sativum*, *Solanum tuberosum*, and *Zea mays*. Using the tblastn tool [23], we searched LAB Transcriptome v6.1 (which contains only laboratory *N. benthamiana* variants) at the http://sefapps02.qut.edu.au/ (accessed on 9 July 2021) database for the homologous sequences based on the protein sequences of the abovementioned RGPs. Next, the obtained *N. benthamiana* sequences were analyzed for correspondence to the putative coordinates in the genome scaffolds. TopHat2 [24] analysis of the ver. 5 genome database (http://sefapps02.qut.edu.au/ (accessed on 10 July 2021)) resulted in the mapping of the sequences of putative RGPs obtained at the previous step to four unique loci in the scaffolds. Therefore, we conclude that *N. benthamiana* contains four distinct *RGP* genes. We designated these genes *NbRGP1–3* and *5* based on the similarity with *AtRGPs 1–5* [6,15] and UDP-arabinopyranose mutases (UAMs) 1–4 from *N. tabacum* [8].

**Figure 1:**
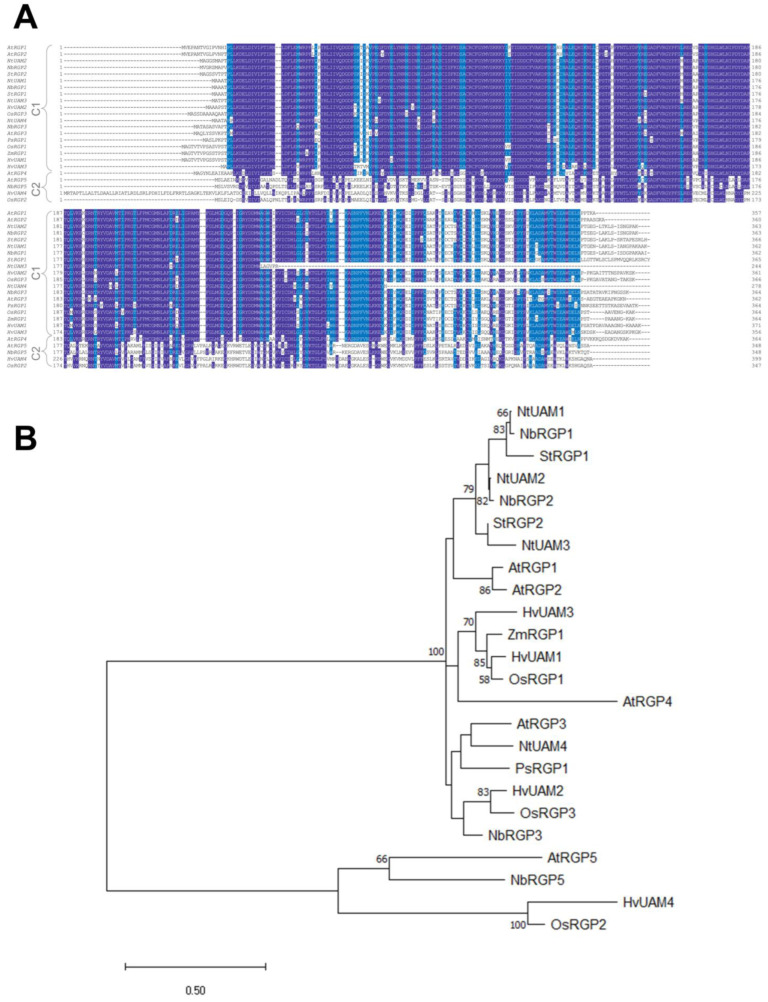
Phylogenetic analysis of the *Nicotiana benthamiana* RGP family. (**A**) Multiple alignment of RGP amino acid sequences from selected species, *N. benthamiana* (NbRGP1–3, 5), *A. thaliana* (AtRGP1–5), *O. sativa* (OsRGP1–3), *H. vulgare* (HvUAM1-4), *P. sativum* (PsRGP1), *S. tuberosum* (StRGP1–2), *N. tabacum* (NtUAM1-4), and *Z. mays* (ZmRGP1), performed using ClustalW. The top 19 sequences are class 1 (C1) RGPs and the last 5 ones are class 2 (C2) RGPs. Amino acid residues common for >80% of analyzed sequences are highlighted in dark blue, whereas positions highlighted in light blue are conserved in >60% of sequences. (**B**) The phylogenetic tree of RGPs listed in (**A**). The tree was obtained using the maximum likelihood method and the model of Lèbre and Gascuel [26]. The percentage of trees in which the associated taxa clustered together is shown next to the branches. The tree is drawn to scale, with branch lengths representing the number of substitutions per site. Bootstrap values for 10,000 replications are given at the branch points. Evolutionary analyses were conducted in MEGA X [27].

To analyze the identified sequences, we used ClustalW [25] to perform multiple alignment of RGPs (UAMs) from *A. thaliana* (AtRGP1–5), *O. sativa* (OsRGP1–3), *H. vulgare* (HvUAM1-4), *P. sativum* (PsRGP1), *S. tuberosum* (StRGP1–2), *N. tabacum* (NtUAM1–4), and *Z. mays* (ZmRGP1) (Figure 1A) and obtained a phylogenetic tree (Figure 1B). Three of the four identified NbRGPs belong to class 1, which are characterized as proteins with UAM activity (except for AtRGP4, which was shown to have no mutase activity thus could be included in class 2 but, in terms of sequence similarity, is the closest one to class 1 [6]). One NbRGP, designated as NbRGP5, together with AtRGP5, OsRGP2, and HvUAM4, belongs to class 2, the members of which are not mutases. NbRGP 1–3 are highly similar, demonstrating from about 87% to 95% identity between each other, whereas NbRGP5 has only about 50% similarity with these three NbRGPs (Figure S1). The analysis of the *N. benthamiana* genome assembly indicated that *NbRGP1*, *2*, *3* genes contain four exons, whereas *NbRGP5* is intronless. The presence of introns was confirmed experimentally: we compared the PCR products that were obtained using genomic DNA or cDNA as a template and a pair of primers corresponding to the 5′ and 3′ ends of the gene (Figure S2). 

**Figure 2:**
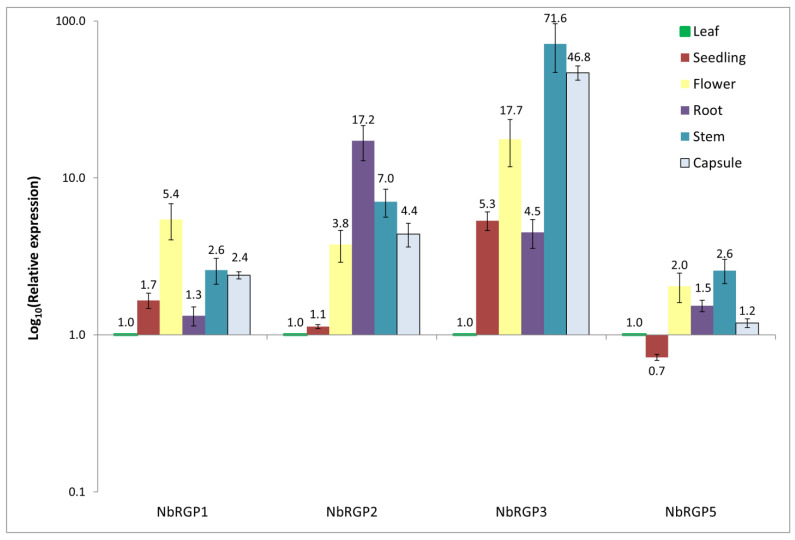
Quantitative RT-PCR analysis of RGP expression in different *N. benthamiana* organs and seedlings. Seedlings were grown for 10 d. Leaves and stems were derived from seven-week-old plants. Flowers were obtained from the same plants one week later, and capsules with seeds were harvested from ten-week-old plants. The levels of expression are normalized to the *PP2A* gene. The plot represents means ± SE of three technical repeats from three biological replicates each.

The expression patterns of *N. benthamiana RGPs* in seedlings and various parts of the plant, such as the roots, stems, leaves, flowers, and capsules, were assessed using quantitative RT-PCR (Figure 2). All four *NbRGPs* are highly expressed in stems. *NbRGP1* and *NbRGP5* are expressed more evenly in all analyzed samples than the others: the difference does not exceed 10 times, whereas the other *NbRGPs* have a more variable pattern of expression within organs. *NbRGP*2 mRNA also accumulates at more or less comparable levels in flowers, roots, stems, and capsules, but root expression prevails. *NbRGP3* expression prevails in stems, capsules, and flowers as compared with leaves. 

### 2.2. Tobacco Mosaic Virus Systemic Infection Affects NbRGPs Expression

**Figure 3:**
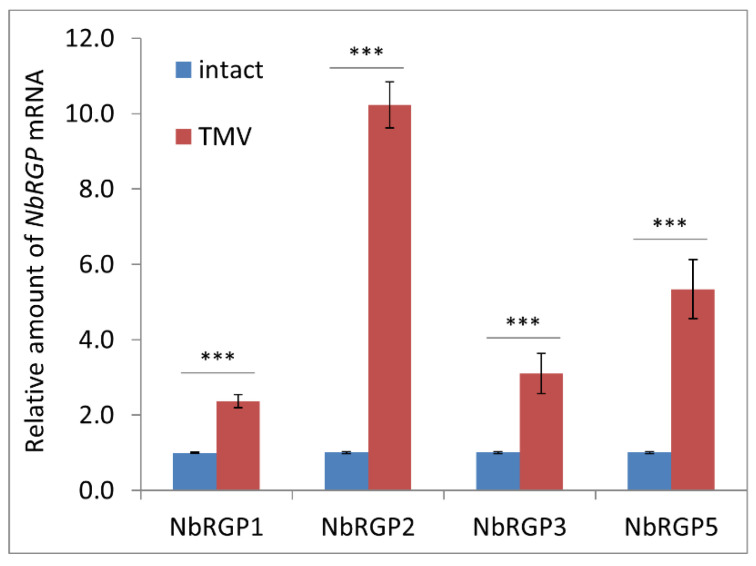
*NbRGP* expression in *N. benthamiana* leaves with systemic TMV infection. The relative amount of *NbRGP* mRNA in leaves with TMV systemic infection as determined by qRT-PCR. The difference between the control (samples from the mock-inoculated plants of the same age) and infected leaves is significant at *p* < 0.001 (Student’s *t*-test) and is marked with ***.

RGPs from different plant species were demonstrated to participate in stress-induced responses to various biotic and abiotic stress factors including viral pathogens. In this paper, we assessed the response of NbRGPs to TMV systemic infection in *N. benthamiana*. To obtain systemic infection, we inoculated the lower leaves of five- or six-week-old plants with TMV. When symptoms of the infection were visible in upper leaves, we confirmed the presence of TMV coat protein in extracts from these leaves: total soluble protein was separated by PAAG electrophoresis followed by Coomassie blue staining (Figure S3). We analyzed *NbRGP1–3*, *5* mRNA level in the leaves with confirmed systemic TMV infection using qRT-PCR (Figure 3) and revealed a significant increase in *NbRGP1–3* and *5* mRNA accumulation compared with the samples taken from the mock-inoculated plants. 

### 2.3. Class 1 NbRGPs Increased Expression Leads to the Suppression of TMV Intercellular Transport and Reproduction

**Figure 4:**
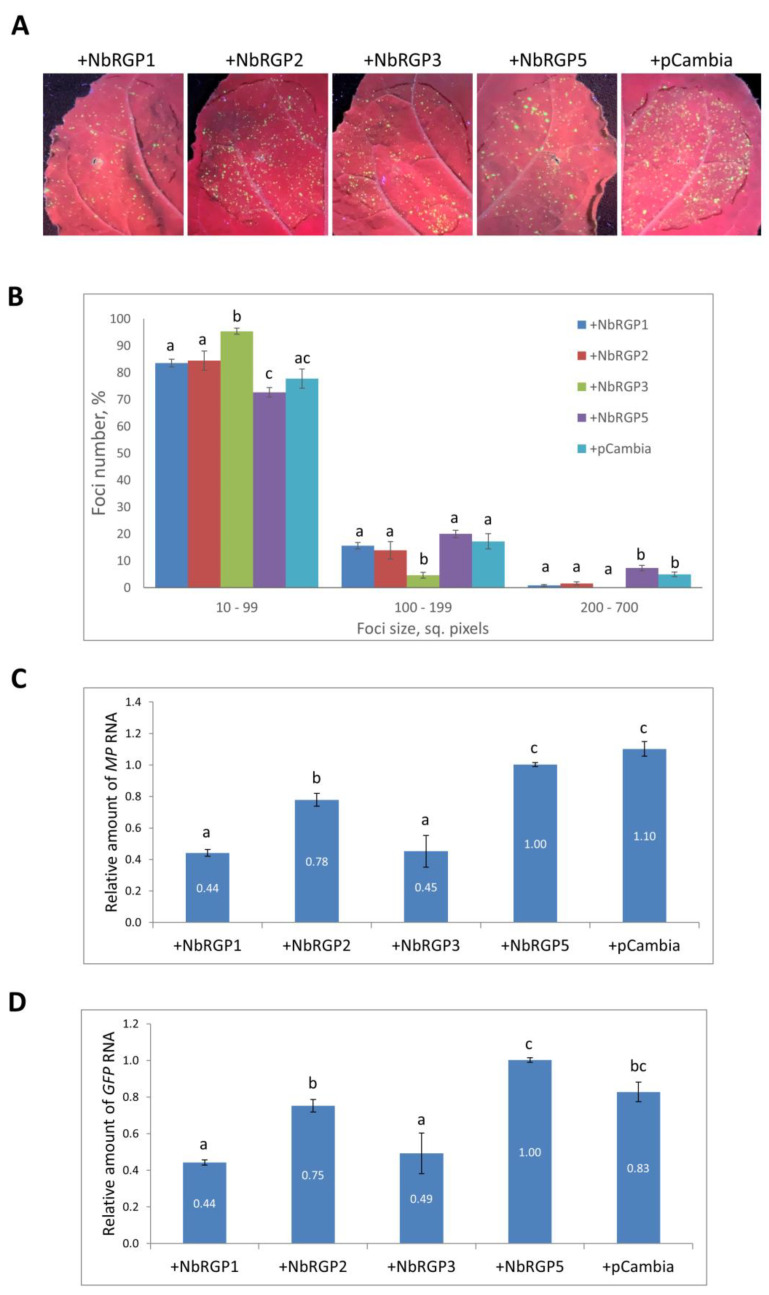
Increased expression of class 1 *NbRGPs* suppresses TMV:GFP reproduction and intercellular spread. (**A**) *GFP*-expressing foci visualization under UV light in *N. benthamiana* leaves four days after co-agroinfiltration with TMV:GFP and each of *NbRGP1–3* and *5*. Combination with pCambia is used as a negative control. (**B**) The percentage of TMV:GFP-expressing foci of different sizes. (**C**,**D**) Relative amounts of MP- (**C**) and GFP-encoding (**D**) RNA in analyzed leaves, quantified using qRT-PCR. Mean values and standard errors are presented in histograms (**B**–**D**). The data were analyzed using ANOVA. Bars without the same letters indicate significant differences according to Tukey’s HSD at *p* < 0.05.

Constitutive expression of *AtRGP2:GFP* had previously been shown to have a negative impact on TMV infection spread in *N. tabacum* (Samsun NN) transgenic plants compared with wild-type plants [15], whereas silencing of *NbRGP1–3* in *N. benthamiana* stimulated TMV intercellular transport [16]. We checked the effect of increased expression of *NbRGP1–3* and *5* on viral intercellular transport using a TMV-based viral vector, TMV:GFP, [28] in which a coat protein gene was replaced with GFP, and assessed the local spread of infection measuring the GFP-expressing foci size. We co-infiltrated *N. benthamiana* leaves with TMV:GFP with each of the *NbRGP*-expressing constructs and pCambia1300 binary vector as negative control. Four days post-inoculation the foci of TMV:GFP infection were visualized under UV light (Figure 4A), and their size was measured using ImageJ software, version 1.15i [29]. The efficiency of agrobacterium-mediated transfection was shown to be similar for all experimental groups based on the foci number. The area of the focus reflects the ability of a viral vector to spread from cell to cell, characterizing the activity of its intercellular transport. *NbRGP5* overexpression did not result in significant changes in TMV:GFP spread, and the distribution of foci of different sizes was comparable with those registered for the negative control (pCambia1300). In the parts of the leaf with *NbRGP1*, *2*, or *3* overexpression, TMV:GFP spread was significantly suppressed compared with *NbRGP5*-expressing areas: the number of smaller foci (10–99 sq. pixels) increased up to 83.5 ± 1.4% (NbRGP1), 84.5 ± 3.6% (NbRGP2), and 95.4 ± 1.1% (NbRGP3), in contrast to only 72.7 ± 1.8% for NbRGP5. The number of larger foci (200–700 sq. pixels) decreased to 0.8 ± 0.3% and 1.6 ± 0.6% for *NbRGP1* and *2*, respectively, versus 7.3 ± 1.1% for *NbRGP5*; no foci of this size were detected in the *NbRGP3*-expressing area (Figure 4B). 

In addition, we assessed the efficiency of viral reproduction in the same infiltrated areas by analyzing the accumulation of RNA containing the genes for movement protein (MP) and GFP produced from the TMV:GFP vector (Figure 4C,D). The levels of accumulation of these RNAs demonstrated that class 1 NbRGP1–3 suppressed viral reproduction but class 2 NbRGP5 did not.

We concluded that only class 1 NbRGPs (not class 2 NbRGP5) adversely affect the development of TMV infection, restraining local transport of the virus and downregulating viral RNA accumulation.

### 2.4. NbRGP1 and 2 Stimulate Plasmodesmata Callose Deposition

**Figure 5:**
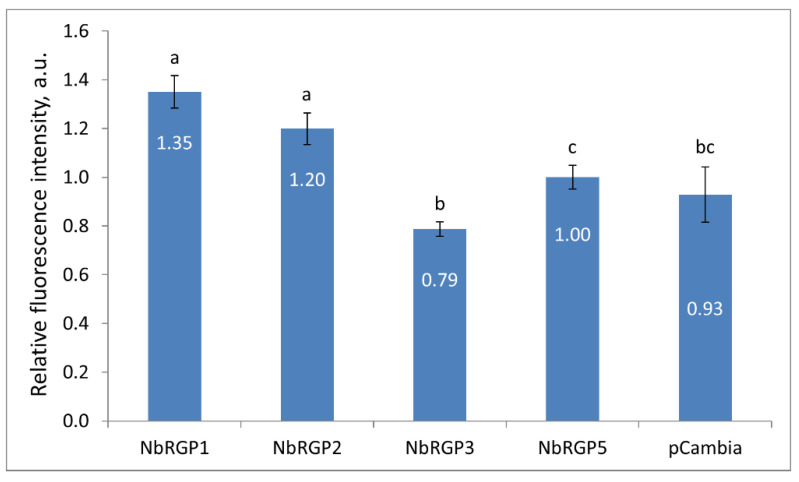
NbRGPs affect Pd callose depositions. Relative Pd callose levels as estimated based on measurement of aniline-blue-stained callose fluorescence intensity in *N. benthamiana* leaves 24 h after agroinfiltration with either of an NbRGP-encoding construct or pCambia. The level of callose in leaves expressing 35S-NbRGP5 was taken as 1. Mean values in arbitrary units (a.u.) and standard errors are presented. The data were analyzed using ANOVA. Bars without the same letters indicate significant differences according to Tukey’s HSD at *p* < 0.05.

We showed that an increased expression of class 1 NbRGPs restricts intercellular viral spread, which could indicate reduced Pd conductivity. The most extensively studied mechanism of Pd regulation involves callose, and numerous studies have shown that callose participates in the regulation of Pd permeability: excessive callose deposition decreases the Pd apertures, whereas a reduction in callose leads to their increase [30,31,32]. Moreover, it was suggested that a AtRGP2-mediated reduction in Pd permeability in mature leaves may be due to partial Pd occlusion caused either by AtRGP2 accumulation in Pd [9] and/or by Pd constriction caused by excessive callose deposition [15]. To check if NbRGPs affect the Pd permeability via a callose-mediated mechanism, we agroinfiltrated *N. benthamiana* leaves with each of 35S-based NbRGP-encoding constructs. Aniline blue staining followed by confocal laser microscopy imaging (Figure S4) allowed quantitative assessment of the amount of callose deposited at Pd, based on the fluorescence intensity [33]. The number and the size of the depositions did not differ between *NbRGP*-overexpressing leaves at 24 hpi, whereas the fluorescence intensity of stained callose elevated in response to *NbRGP1* and *2* increased the expression by 35% and 20%, respectively, compared with areas with *NbRGP5* overexpression (Figure 5). As we did not detect any statistically significant differences between areas agroinfiltrated with 35S-NbRGP5 and negative control (pCambia1300) the values were normalized to NbRGP5. Surprisingly, 35S-NbRGP3 expression led to a ~20% decrease in callose deposition rather than to an increase in it compared with 35S-NbRGP5.

We concluded that NbRGP1 and 2 negatively regulate Pd permeability for viral infection via a callose-mediated mechanism.

### 2.5. NbRGP1 Binds TMV MP In Vitro

The primary function of TMV MP is to mediate viral RNA intracellular transport to Pd and via Pd to the neighboring cells, thereby enabling local spread of viral infection. We suggested that, in addition to the callose-dependent mechanism by which NbRGP1 and 2 restrict viral spread, there could be an alternative mechanism that involves direct interaction between NbRGPs and MP, preventing the proper functioning of the MP during viral infection. We chose NbRGP1 for further interaction and localization studies because, according to the results described above, both NbRGP1 and 2 have similar features and the same effect on viral infection. Moreover, their amino acid sequences are characterized by ~95% identity.

**Figure 6:**
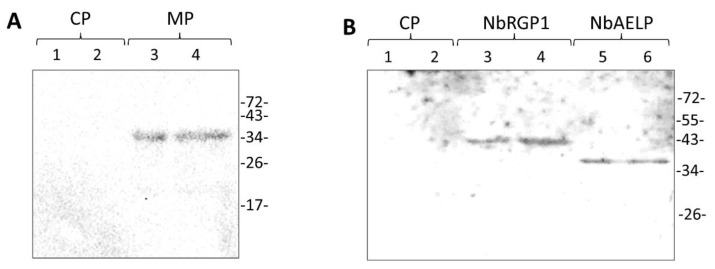
NbRGP1 and MP interact in vitro in the renatured blot overlay binding assay. (**A**) A membrane with immobilized TMV CP as the negative control (lane 1–1 μg, lane 2–2 μg) and MP (lane 3–1.5 μg, lane 4–3 μg) was incubated in a renaturing buffer with NbRGP1. The membrane was further probed with antibodies against NbRGP1. (**B**) A membrane with immobilized TMV CP as the negative control (lane 1–1.5 μg, lane 2–3 μg), NbRGP1 (lane 3–3 μg, lane 4–4 μg), and NbAELP as the positive control (lane 5–1.5 μg, lane 6–2 μg) was incubated in the renaturing buffer with TMV MP. The membrane was further probed with antibodies against MP.

First, we checked whether NbRGP1 and MP interact in vitro using the renatured blot overlay binding assay. For this purpose, we immobilized recombinant 6His-MP on the membrane and incubated the membrane with the solution containing 6His-NbRGP1. Figure 6A shows that NbRGP1 specifically binds to the immobilized MP as the signal is detected by the antibodies against NbRGP1 in the region that corresponds to MP. No signal was detected in the lane with immobilized TMV coat protein (CP), which was used as a negative control. This reciprocal blot (Figure 6B), with 6His-NbRGP1 immobilized on the membrane and 6His-MP in the solution, confirms this result: the signal from MP-specific antibodies is detected in the band that corresponds to 6His-NbRGP1 but not in the lane with the negative control (TMV CP). *N. benthamiana* aldose-1-epimerase-like protein (NbAELP), previously shown to interact with MP [34], was used as a positive control. 

Therefore, we conclude that NbRGP1 and MP specifically bind in vitro.

### 2.6. NbRGP1 Interacts with TMV MP In Vivo 

**Figure 7:**
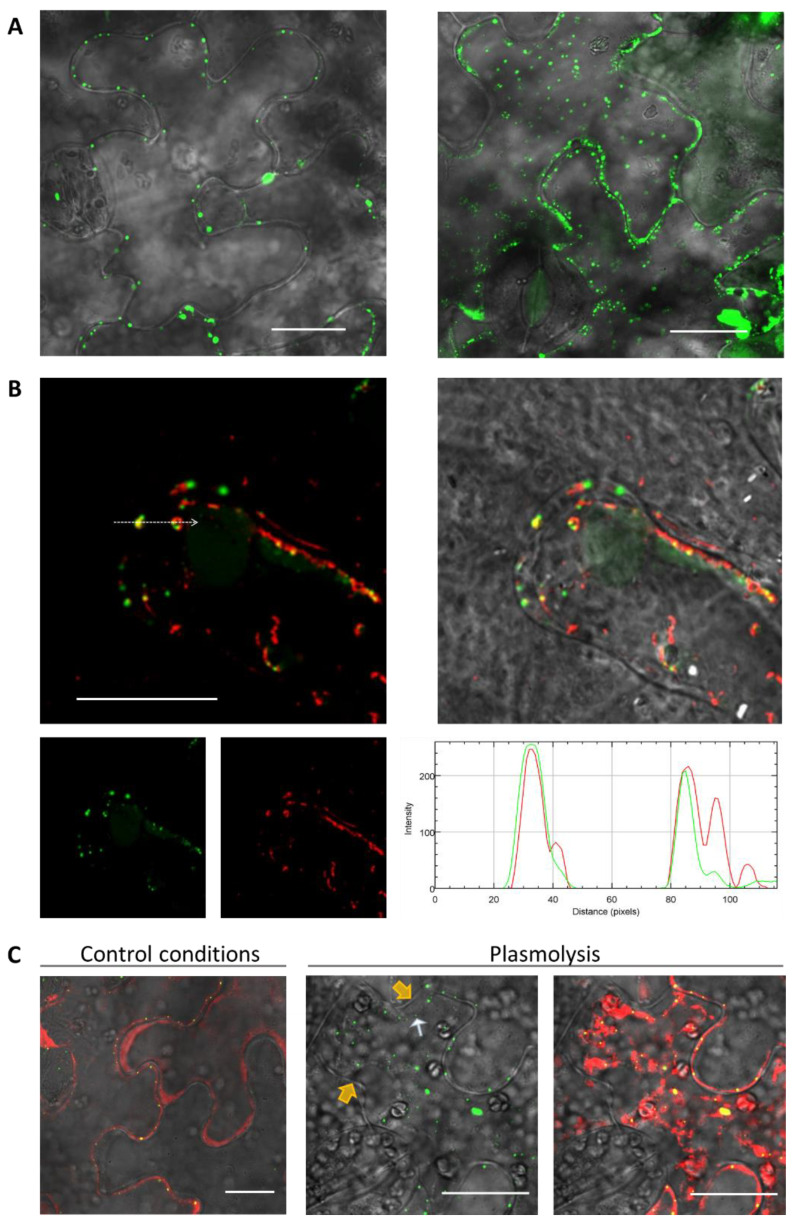
NbRGP1 subcellular localization in *N. benthamiana* leaves. (**A**) Images of NbRGP1:GFP expressing epidermal cells of *N. benthamiana* leaves 3 dpi were obtained using confocal fluorescence microscopy. Optical sections taken through the middle of the cell (**left**) and cortical cytoplasm (**right**). Overlay of single confocal images of the GFP and bright-field channels. (**B**) NbRGP1:GFP and AtGONST4:RFP co-localization in epidermal cells of *N. benthamiana* leaves. Overlay of single confocal images of RFP (red), GFP (green), and bright-field channels. An arrow marks the segment along which the profile of relative fluorescence intensity (green line for GFP and red line for RFP) is plotted. (**C**) NbRGP1:GFP subcellular localization in epidermal cells of *N. benthamiana* leaves after joint infiltration with agrobacteria containing 35S-NbRGP1:GFP or 35S-RFP plasmid under control conditions (**left**) and after plasmolysis (**right**). Overlay of single confocal images of the RFP, GFP, and bright-field channels. The cell wall is marked with yellow arrowheads, and white arrowheads demarcate NbRGP1:GFP in the protoplast retracted after plasmolysis. Bars = 20 μm. All mixtures for infiltration were supplemented with agrobacteria containing plasmid for expression of p19 silencing suppressor of tomato bushy stunt virus.

To check the hypothesis regarding NbRGP and MP interactions in vivo, we first analyzed NbRGP1 intracellular localization. We agroinfiltrated *N. benthamiana* leaves with 35S-NbRGP1:GFP. Confocal laser scanning microscopy revealed that NbRGP1:GFP demonstrated punctate distribution of a fluorescent signal at the cell periphery (Figure 7A, left).

However, an optical section taken through the upper cortical cytoplasm near the cuticle where there are no Pd demonstrates punctate GFP fluorescence, probably corresponding to Golgi vesicles or other vesicle-like structures (Figure 7A, right). According to the previous reports [6,9,22], AtRGP1 and 2 are detected in association with the Golgi apparatus. We obtained the genetic construct 35S-AtGONST4:RFP, encoding Golgi marker AtGONST4 [35] fused to RFP, and co-expressed it with 35S-NbRGP1:GFP to check if the latter was Golgi associated. Figure 7B demonstrates that NbRGP1 co-localizes with the AtGONST4 Golgi marker, indicating that NbRGP1 is associated with Golgi vesicles. At the same time, peripheral NbRGP1:GFP puncta that could be Pd were also visible. We stained Pd callose with aniline blue in areas expressing NbRGP1:GFP. The signal from GFP and callose mainly did not overlap (Figure S5). However, few signals co-localized. Thus, we could not exclude that a portion of NbRGP1 has Pd localization based on these results. To clarify how NbRGP1 is distributed in the cell we used a plasmolysis technique. This approach allows distinguishing of Pd-localized proteins from cytoplasmic and membrane proteins. We performed plasmolysis of cells expressing 35S-NbRGP1:GFP together with 35S-RFP for cytoplasm staining. NbRGP1:GFP localized to the cytoplasm in plasmolyzed cells, demonstrating no plasmodesmal or cell wall localization (Figure 7C).

**Figure 8:**
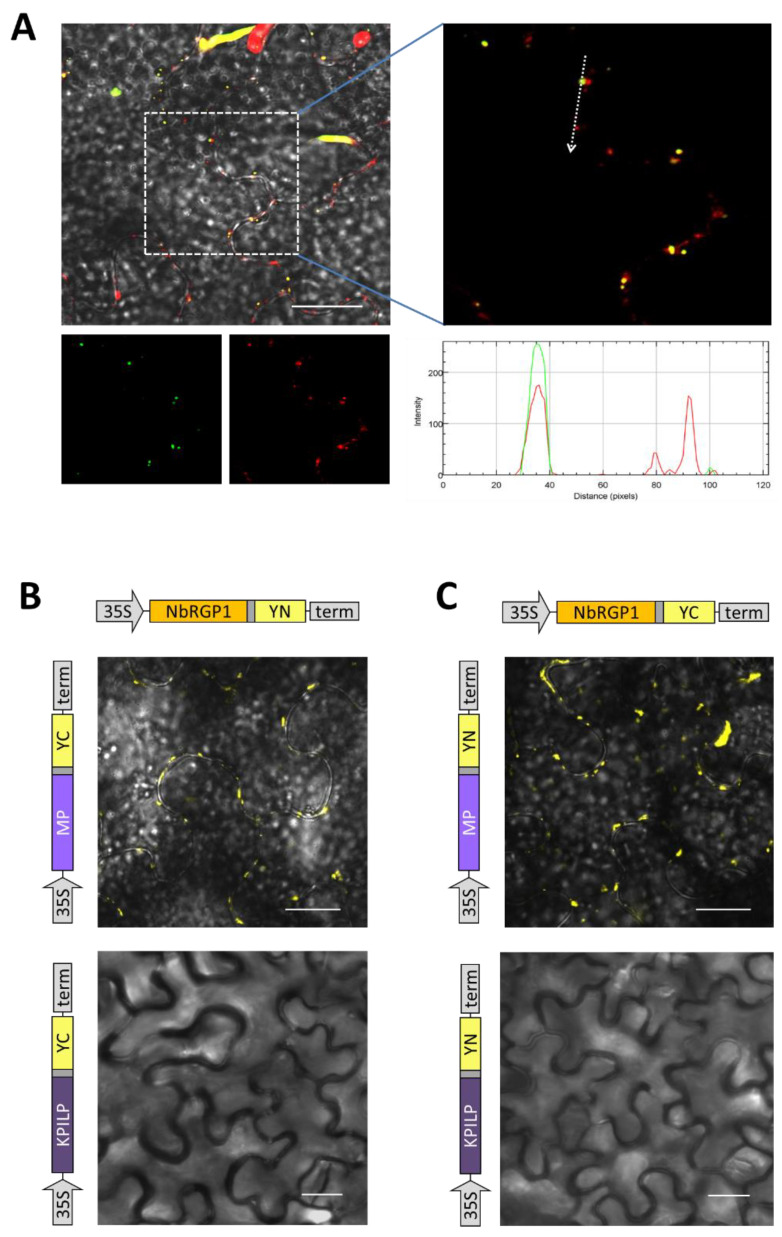
NbRGP1 co-localizes and interacts with TMV MP. (**A**) Subcellular localization of NbRGP1:GFP and MP:RFP in epidermal cells of *N. benthamiana* leaves three days after joint agroinfiltration with 35S-NbRGP1:GFP and 35S-MP:RFP. Overlay of single confocal images of the RFP, GFP, and bright-field channels. An arrow on the enlarged image of the cell co-expressing 35S-NbRGP1:GFP and 35S-MP:RFP marks the segment along which the profile of relative fluorescence intensity (green line for GFP and red line for RFP) is plotted. (**B**,**C**) YFP fluorescence analyzed using fluorescent microscopy 3 dpi days after infiltration of *N. benthamiana* leaves with pairs of agrobacteria containing plasmids for (**B**) expression of 35S-NbRGP1:YN and 35S-MP:YC (**upper panel**) and (**C**) 35S-NbRGP1:YC and 35S-MP:YN (**upper panel**). 35S-KPILP:YN and 35S-KPILP:YC are used as negative control ((**B**,**C**), **lower panel**). Bars = 20 μm. All mixtures for infiltration were supplemented with agrobacteria containing plasmid for expression of the p19 silencing suppressor of the tomato bushy stunt virus.

To test the hypothesis about NbRGP and MP interactions, we performed co-expression of 35S-NbRGP1:GFP and 35S-MP:RFP and detected a partial co-localization of GFP and RFP signals in intracellular punctate structures (Figure 8A). To understand whether these proteins interact we used a bimolecular fluorescence complementation (BiFC) approach. We obtained genetic constructs encoding NbRGP1 or MP fused with the N- or C-terminal part of YFP (Figure 8B,C). Here, we used *N. benthamiana* Kunitz peptidase inhibitor-like protein (KPILP) as a negative control. KPILP, similar to NbRGPs, was recently demonstrated to be upregulated in response to TMV infection [36]. However, in contrast to NbRGPs, KPILP stimulates the development of viral infection, facilitating its intercellular spread. Co-expression of 35S-KPILP:YN or 35S-KPILP:YC with 35S-NbRGP1:YC or 35S-NbRGP1:YN, respectively, did not lead to the restoration of YFP chromophore (Figure 8B,C, bottom panels). However, for pairs encoding NbRGP1 or MP we observed YFP fluorescence in the infiltrated areas (Figure 8B,C, top panels), indicating that NbRGP1 and MP could interact in vivo. 

## 3. Discussion

Reversibly glycosylated polypeptides are found in numerous plant species, both dicots and monocots, as well as in algae, mosses, spore plants, etc. [6,10,37]. They play an important role at various stages of plant growth and development, contributing to cell wall formation, pollen development, response to different stresses, plant–pathogen interactions, and intercellular transport regulation. Attention to this protein family is related mainly to their UDP-Arap mutase activity, as Arap–Araf interconversion is indispensable for plant development [6,10,37,38]. 

We identified four *RGP* genes in the *N. benthamiana* genome assembly. As alignment with other known RGPs and a constructed phylogenetic tree show, NbRGPs 1–3 and NbRGP5 can be classified as class 1 and class 2 RGPs, respectively. We obtained the expression pattern of identified *NbRGPs* using qRT-PCR (Figure 2). According to our results, *NbRGP5* expression is evenly distributed in all analyzed organs and is lower in seedlings, which is consistent with the results from the Version 6 Gene expression Atlas (https://sefapps02.qut.edu.au/atlas/tREX6.php (accessed on 8 August 2021)) based on the microarray data (Figure S6). However, for *NbRGP1-3*, the Gene Expression Atlas demonstrates prevailing root expression, whereas our data does not fully confirm such distribution: high root expression is detected for *NbRGP2–3* rather than *NbRGP1*. This discrepancy between the qRT-PCR result and microarray data for *NbRGP1* and *2* could be explained by the high sequence similarity of these two genes, due to which the assembly and distribution of the microarray-based data might be not fully correct. In leaves, all *NbRGPs* are fairly weakly expressed, and *NbRGP1* and *5* are represented mainly in leaves compared with other organs. The observed expression pattern of *NbRGPs* (Figure 2) resembles the previously obtained one for *AtRGPs* [22]. 

NbRGPs 1–3 are highly similar to the recently identified UAMs from *N. tabacum* (Figure 1), for which UAM activity was confirmed in vitro [8]. This suggests that NbRGPs 1–3 could also be classified as UAMs. However, there is another equally important aspect of RGP function, namely their role in the development of viral infection [9,15,16,20]. We analyzed *NbRGP* mRNA accumulation levels in *N. benthamiana* plants with systemic TMV infection and revealed that viral infection induces a significant increase in the expression of all four *NbRGPs* (Figure 3). 

Previously, constitutively expressed *AtRGP2:GFP* in *N. tabacum* was demonstrated to limit TMV local and systemic spread [15]. In this study, we assessed the role of NbRGPs in the development of TMV infection in *N. benthamiana* plants. We created a model system using a TMV-based TMV:GFP viral vector to imitate infection and performed co-expression of TMV:GFP with each of the identified *NbRGPs* in leaves. Visualizing infection foci under UV light, we assessed the efficiency of intercellular spread of the virus, measuring zones with GFP fluorescence. We found out that all three class 1 RGPs suppress intercellular transport of the virus as compared with NbRGP5 and control, an empty binary vector (pCambia1300). Viral replication manifesting itself in accumulation of the viral RNA encoding MP and GFP was also reduced when there was increased expression of class 1 *NbRGPs* rather than *NbRGP5*. Therefore, we showed that the increased expression of class 1 *NbRGPs* provides protection from viral infection, which is consistent with the data obtained by Burch-Smith et al., who showed that class 1 *NbRGP* silencing accelerated the development of systemic TMV infection, whereas a TMV-based viral vector demonstrated more extensive spread in those plants [16]. 

Our research into the mechanism of antiviral action of class 1 NbRGPs stemmed from the following: firstly, a well-known fact that RGPs are capable of increasing callose deposition around Pd [15] and secondly that RGPs can be localized in Pd, creating a physical barrier for the intercellular transport of macromolecules [9,15,21]. Thirdly, when RGPs are localized in Pd they can interact with TMV MP and inhibit its function, i.e., intercellular transport of viral genetic material. Indeed, according to our data, elevated *NbRGP1* and *2* expression leads to an increase in Pd callose levels by 35% and 20%, respectively, as compared with *NbRGP5* (Figure 5). As far as NbRGP3 is concerned, the results were contradictory. The increased *NbRGP3* expression suppressed TMV reproduction and intercellular transport; however, it caused a reduction in Pd callose level. This could be explained either by the fact that NbRGP3 needs to form complexes with other NbRGPs to fulfil its function or by the spatiotemporal features of its expression, i.e., the fact that normally in plants it is expressed and functions presumably not in leaves but other organs or at other stages of development. Thus, in our experimental system its expression in leaves is ectopic. For instance, research into *AtRGP3*‘s expression pattern revealed its transient but strong expression during seed development [6]. The authors suggest that AtRGP3 might be involved in Ara supply for the growing cell wall of the developing embryo, delivered via the endosperm. When we analyzed the expression of all four *NbRGPs* in response to the systemic TMV infection, we revealed *NbRGP3* upregulation in leaves. Therefore, the mechanism for its suppressing impact on TMV reproduction and intercellular transport calls for further studies. 

To study the intracellular localization, we chose NbRGP1, which had a high degree of amino acid sequence identity (94%) with NbRGP2 and with AtRGP1 and AtRGP2 (92% and 90% respectively). Additionally, the NbRGP1 nucleotide sequence has 97% similarity with *N. tabacum* UAM1, whereas their amino acid sequences are almost identical (99%) [8]. Our results revealed punctate distribution of NbRGP1:GFP at the cell periphery and partial co-localization with MP (Figure 8A), which could be suggested to correspond to the Golgi apparatus that is characteristic of the RGPs of other species [4,5,6,9,22]. We also detected aggregates of NbRGP1:GFP in the cytoplasm similar to those previously described [4,6,22]. As regards to Pd localization, we did not find NbRGP1 in Pd of *N. benthamiana* leaves (Figure 7C). We have demonstrated that NbRGP1:GFP co-localized with the Golgi marker AtGONST4 (Figure 7B), which is in line with previously published results for other RGPs. Several research groups have showed that different RGPs are associated with Golgi membranes and distributed in the cytoplasm [2,4,6,19,39]. However, it could not be excluded that NbRGP1 interacting with MP is potentially relocalized to Pd, serving there as a mechanical obstacle for intercellular transport. Sagi and colleagues [9] put forward such a hypothetical model for RGP-mediated Pd permeability reduction. We used two approaches that convincingly confirmed the interaction between these two proteins in vitro and in vivo. We demonstrated interaction between NbRGP1 and MP in vitro using the overlay assay technique (Figure 6). Furthermore, using the BiFC system, we demonstrated that both reciprocal pairs of NbRGP1 and TMV MP translational fuses formed a pair, with the restoration of YFP fluorescence in *N. benthamiana* leaves, indicating NbRGP1/MP interactions in vivo (Figure 8B,C). NbRGP1 and TMV MP likely interact on Golgi membranes. In this study, we did not investigate in detail the intracellular localization of NbRGP5 or its ability to interact with either TMV MP or other class 1 NbRGPs. However, we showed that NbRGP5 does not affect callose depositions and does not suppress TMV intercellular transport. Our phylogenetic analysis also demonstrated that NbRGP5 belongs to class 2, which lack mutase activity. Regarding the biological role of NbRGP5, we can assume that NbRGP5 participates in the formation of heteroprotein complexes with enzymatically active NbRGPs, as it was shown for RGPs of other species [40], which may determine its stabilizing function in these interactions. This is why its expression increases in response to the viral infection without a direct negative impact on the virus. Therefore, the role of class 2 RGPs such as NbRGP5 in the development of viral infection is to be further investigated.

**Figure 9:**
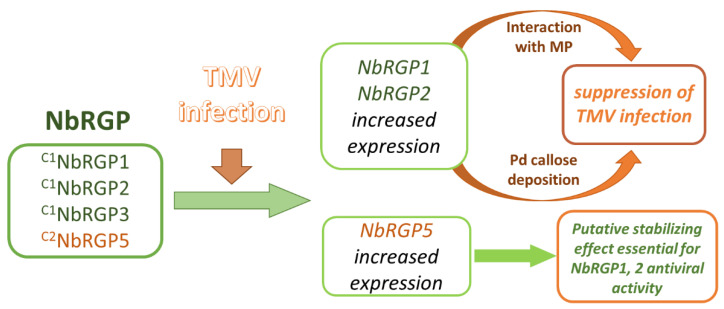
A hypothetical model illustrating the role of NbRGPs in the development of TMV infection in *N. benthamiana*.

All our results are in line with the hypothesis accounting for the protective role of NbRGPs in interaction between *N. benthamiana* and TMV (Figure 9) and are consistent with the previously reported data on RGP antiviral action [15,16]. The viral infection activates the expression of all four *NbRGPs*. We assume that the antiviral mechanism consists of blocking intercellular transport of the virus: firstly, via the callose-mediated mechanism and secondly, via a direct interaction with MP, which probably interferes with MP transport function. 

In this article, we presented convincing evidence that NbRGP1 and 2 stimulate callose deposition and NbRGP1 interacts with TMV MP. Being very similar in terms of amino acid and nucleotide sequences, NbRGP1 and 2 could have the same function in a cell and act in a similar way by stimulating callose deposition and potentially blocking MP function by interacting with it. Although we do not investigate the intracellular localization of NbRGP2 and its ability to interact with MP, its antiviral effect seems obvious and results are presented that demonstrate and confirm this function.

Therefore, based on the results of this study, we can conclude that class 1 NbRGP1 and NbRGP2, induced in response to TMV infection, suppress the intercellular transport of the virus and its reproduction. NbRGP1 stimulates the “closing” of plasmodesmata via the callose-mediated mechanism and interacting with TMV MP probably inhibits the intercellular transport of viral RNA. To summarize, we can state that NbRGPs play the role of antiviral cellular factors.

## 4. Materials and Methods

### 4.1. Plant Growth Conditions

Wild-type *N. benthamiana* plants were grown in soil in a controlled environment chamber under a 16 h/8 h day/night cycle.

### 4.2. Plasmid Constructs

The sequences encoding NbRGP1, 2 and 5 were amplified from *N. benthamiana* cDNA using the F1/R1, F2/R2, and F5/R5 primer pairs, respectively. Acc65I and SalI recognition sites at the 5′ and 3′ ends were introduced in the resulting fragment. NbRGP3 sequence was amplified with the F3/R3 primer pair. The resulting PCR product was flanked with NruI and SalI recognition sites. The PCR products were digested with the Acc65I-SalI or NruI-SalI restriction enzymes and cloned into the pCambia1300-based vector containing the 35S promoter and terminator (pCambia-35S) and digested with Acc65I/SalI or NruI/SalI. Therefore, we obtained 35S-NbRGP1, 35S-NbRGP2, 35S-NbRGP3, and 35S-NbRGP5 genetic constructs. The 35S-NbRGP1:GFP construct was obtained as follows. Using the F1/R4 primer pair, a fragment encoding NbRGP1 without a stop codon and flanked by Acc65I/BamHI recognition sites was obtained. The fragment encoding GFP without a start codon and flanked by BamHI/SalI recognition sites was produced using F9/R9 pair of primers. The NbRGP1 fragment without a stop codon, together with a GFP fragment, was ligated into a pCambia-35S vector digested with Acc65I/SalI enzymes. 

To obtain plasmids for the BiFC system, the fragment encoding 154 YFP N-terminal amino acids (designated YN) and the fragment encoding 86-aa C-terminal part of YFP (YC) were amplified using the F4/R11 and F6/R12 primer pairs, respectively. Both fragments contained an ApaI recognition site instead of a start codon and SalI downstream of the stop codon. 35S-NbRGP1:YN and 35S-NbRGP1:YC were constructed by substitution of YN or YC fragments for GFP in 35S-NbRGP1:GFP plasmid using ApaI and SalI sites. 

Sequences encoding AtGONST4 (NM_122005.4) without a stop codon were amplified from *A. thaliana* Col1 cDNA using the F11/R14 primer pairs. The fragment encoding RFP without a start codon and flanked by BamHI/SalI recognition sites was produced using F12/R15 pair of primers, respectively. The AtGONST4 PCR product was digested with Acc65I/BamHI and together with RFP fragment flanked with BamHI/SalI inserted into pCambia-35S via Acc65I and SalI sites resulting in 35S-AtGONST4:RFP.

Plasmids encoding MP translational fuses with RFP and YN or YC were obtained using the same algorithm. The TMV MP fragment was produced using a F7/R13 primer pair. The PCR product was digested with Acc65I/BamHI enzymes and cloned together with a fragment encoding the corresponding fluorescent tag into pCambia-35S via Acc65I and SalI sites resulting in the set of constructs: 35S-MP:YN/YC and 35S-MP:RFP.

Plasmids encoding KPILP translational fuses with YN or YC were obtained by amplification of the KPILP fragment using the F13/R16 pair of primers with Acc65I/BamHI flanking sites and 35S-NbKPILP(ACG) plasmid [41] as a template. The PCR product was digested with the Acc65I-BamHI restriction enzymes and cloned into the 35S-NbRGP1:YN/YC plasmids replacing the NbRGP1 fragment.

To obtain a plasmid for NbRGP1 recombinant protein production in *E.coli* cells, a PCR fragment of NbRGP1 without a start codon was obtained with the F8/R1 primer pair and Acc65I and SalI recognition sites at the ends of the resulting fragment. The PCR product was digested with the Acc65I-SalI restriction enzymes and cloned into the pQE-30 (QIAGEN, Netherlands) vector containing the 6xHis-tag at the N-terminus and digested with Acc65I/SalI. Therefore, we obtained a 6xHis-NbRGP1 genetic construction.

GeneBank acc. numbers for NbRGPs: *NbRGP1*, LR961922; *NbRGP2*, OM056463; *NbRGP3*, OM056464; *NbRGP5*, OM056465.

The oligonucleotide sequences are listed in Table S1.

### 4.3. Agroinfiltration Experiments

*A. tumefaciens* strain GV3101 was transformed with individual binary constructs and grown at 28 °C in LB medium supplemented with 50 mg/L rifampicin, 25 mg/L gentamycin, and 50 mg/L carbenicillin/kanamycin. An *Agrobacterium* overnight culture was diluted in an agrobuffer containing 10 mM MES (pH 5.5) and 10 mM MgSO_4_ and adjusted to a final OD_600_ of 0.1. In experiments with viral vector spread, the final OD_600_ for TMV:GFP was 0.005, which allowed to obtain individual transformed cells within the infiltrated area. Agroinjection was performed on almost fully expanded *N. benthamiana* leaves that were still attached to the intact plant. A bacterial suspension was infiltrated into the leaf tissue using a 2 mL syringe, after which the plants were grown under greenhouse conditions at 24 °C with a 16 h/8 h light/dark photoperiod.

### 4.4. Plasmolysis

*N. benthamiana* leaves were infiltrated with a 0.25 M mannitol solution three days after agroinfiltration. About 5–10 min after mannitol solution injection, leaf sections were excised and examined using confocal microscopy. 

### 4.5. Aniline Blue Callose Staining and Quantification

To visualize Pd-located callose, *N. benthamiana* leaves transiently expressing 35S-NbRGPs, as well as control leaves, were infiltrated with aniline blue solution (0.1% aniline blue (Sigma Aldrich, Burlington, VT, USA) in 0.01 M K_3_PO_4_ at pH 12. Then, the leaves were incubated in the dark at room temperature for 15 min before imaging using a Nikon C2 laser scanning confocal microscope. Quantification of the stained callose fluorescence was performed as described by Zavaliev and Epel [33].

### 4.6. GFP, YFP, and RFP Imaging

GFP fluorescence in the inoculated leaves was monitored by illumination with a handheld UV (366 nm) source. The TMV:GFP foci were analyzed at 3 dpi. YFP fluorescence was detected using an AxioVert 200M microscope (Carl Zeiss, Jena, Germany) equipped with AxioCam MRc digital camera. GFP- and RFP-containing fusion proteins were imaged with Nikon C2 confocal laser scanning microscope. The excitation and detection wavelengths for GFP and YFP were 487 nm and 525 nm, respectively; the excitation and emission wavelengths for RFP were 561 nm and 625 nm, respectively. The intracellular distribution of fluorescent proteins was imaged 72 h after infiltration.

### 4.7. Genomic DNA Extraction

*N. benthamiana* mature leaves were frozen in liquid nitrogen and ground into a fine powder. Genomic DNA was extracted using Diatom DNA Prep kit (Galart-Diagnosticum, Moscow, Russia) in accordance with the manufacturer’s protocol.

### 4.8. RNA Extraction and cDNA Synthesis

The total RNA was extracted from plant tissues using TriReagent (MRC, Houston, TX, USA) in accordance with the manufacturer’s instructions. The RNA concentration was determined using a Nanodrop ND-1000 spectrophotometer (Isogen Life Sciences, Utrecht, The Netherlands). For the synthesis of first-strand cDNA, 0.1 mg of random hexamers and 0.1 mg of oligo-dT primer were added to 2 µg of total RNA to obtain cDNA by reverse transcription performed using Superscript II reverse transcriptase (Invitrogen, Waltham, MA, USA) in accordance with the manufacturer’s protocol.

### 4.9. Quantitative Real-Time PCR (qRT-PCR)

Quantitative real-time PCR was carried out using the iCycler iQ real-time PCR detection system (Bio-Rad, Hercules, CA, USA). Target genes were detected using sequence-specific primers (Table S2) and Eva Green master mix (Syntol, Moscow, Russia) in accordance with the manufacturer’s instructions. Reference genes were detected using the primers to the 18S rRNA gene and the protein phosphatase 2A gene (PP2A). Each sample was run in triplicate, and a non-template control was added to each run. A minimum of five biological replicates were performed. The qRT-PCR results were evaluated using the Pfaffl algorithm [42].

### 4.10. Renatured Blot Overlay Binding Assay

Recombinant 6His-NbRGP1 protein was produced in *Escherichia coli* (strain SG13009) and purified via a Ni-NTA affinity chromatography. Briefly, the cells were transfected with the pQE30-based 6His-NbRGP1 construct. An *E. coli* overnight culture was diluted 10 times with liquid LB medium supplemented with kanamycin 25 mg/L and ampicillin 100 mg/L and grown at 37 °C to OD_600_ = 0.6. After addition of isopropyl-β-D-1-thiogalactopyranoside to a final concentration of 1 mM, the culture was incubated for four hours at 37 °C. *E. coli* cells were collected by centrifugation at 4000× *g* for 20 min at 4 °C. 6His-NbRGP1 recombinant protein was purified from the obtained cells by metal-chelate affinity chromatography on Ni-NTA agarose in accordance with “The QIAexpressionist™” handbook protocol. 

The renatured blot overlay assay was performed in accordance with [43], with modifications. Briefly, the analyzed recombinant 6His-NbRGP1 or 6His-MP were resolved in 10% SDS-PAAG and transferred onto a polyvinylidene difluoride membrane (GE Healthcare, Chicago, IL, USA). The membrane was incubated in buffer A (30 mM Tris–HCl pH 7.4, 0.05% Tween-20) for 15 min to remove residual SDS. For immobilized protein denaturation, the membrane was incubated in a denaturing buffer (7 M guanidine hydrochloride, 2 mM EDTA, 50 mM DTT, 50 mM Tris–HCl pH 8.0) for two hours. After the membrane was washed in tTBS (140 mM NaCl, 30 mM Tris–HCl pH 7.4, 0.1% Tween-20), it was incubated overnight at +4 C⁰ in the renaturing buffer (140 mM NaCl, 10 mM Tris–HCl pH 7.4, 2 mM EDTA, 0.05% milk, 0.1% Tween-20, 2 mm DTT). After renaturation of the immobilized protein, the membrane was incubated in the renaturing buffer, supplemented with the potential partner protein added to a concentration of 10 μg/mL for 90 min. Then, the membrane was rinsed in tTBS and incubated in the blocking solution (2.5% powdered skim milk in tTBS) for 1 h. Next, the membrane was incubated in tTBS supplemented with 0.5% skim milk and rabbit polyclonal antibodies against the protein that was in the solution in the previous step—either 6His-NbRGP1 or 6His-MP (Almabion, Voronezh, Russia). Anti-rabbit antibodies conjugated to horseradish peroxidase (Imtek, Moscow, Russia) were used as secondary antibodies. The bands were visualized using a chemiluminescence ECL kit (GE Healthcare, Chicago, IL, USA) and ChemiDoc XRS+ imaging system (Bio-Rad, Hercules, CA, USA).

### 4.11. Statistical Analysis

The data were analyzed either by Student’s *t*-test or by one-way ANOVA, as indicated in figure captions. The significance of difference between groups was assessed using Tukey’s honestly significant difference (HSD) test at *p* < 0.05 level or Student’s *t*-test. In all histograms, *y*-axis error bars represent the standard error of the mean values.

### 4.12. Homology Search and Phylogenetic Analysis

The homology search was conducted using the amino acid sequences for RGP proteins from the UniProt database (https://www.uniprot.org/ (accessed on 9 July 2021)) for the following species: *A. thaliana* (AtRGP1, Q9SRT9; AtRGP2, Q9LFW1; AtRGP3, O22666; AtRGP4, Q9LUE6; AtRGP5, Q9FFD2), *O. sativa* (OsRGP1, Q8H8T0; OsRGP2, Q7FAY6; OsRGP3, Q6Z4G3), *H. vulgare* (HvUAM1, A0A0U2GJJ3; HvUAM2, A0A0U2GJL1; HvUAM3, A0A0U2GJ84; HvUAM4, A0A0U2GJM5), *P. sativum* (PsRGP1, O04300), *S. tuberosum* (StRGP1, Q9SC19; StRGP2, Q8RU27), and *Z. mays* (ZmRGP1, P80607). Multiple alignment was generated using ClustalW [25]. The phylogenetic tree was obtained using the maximum likelihood method and the model of Lebre and Gascuel [26]. The tree is drawn to scale, with branch lengths measured in the number of substitutions per site. Bootstrap values are calculated for 10,000 replications. Evolutionary analyses were conducted in MEGA X [27].

## Data Availability

The original contributions presented in the study are included in the article/Supplementary Material.

## Supplementary Materials

The supporting information can be downloaded at: https://www.mdpi.com/article/10.3390/ijms241612843/s1.
