# Supplementary material for: Nicotiana benthamiana Class 1 Reversibly Glycosylated Polypeptides Suppress Tobacco Mosaic Virus Infection
Source: Int J Mol Sci. 2023 Aug 16;24(16):12843. doi: 10.3390/ijms241612843 (PMC10454303; doi:10.3390/ijms241612843)
Supplement: Supplementary file 1 [file ijms-24-12843-s001.zip › ijms-2490711-supplementary.pdf]

Supplementary material

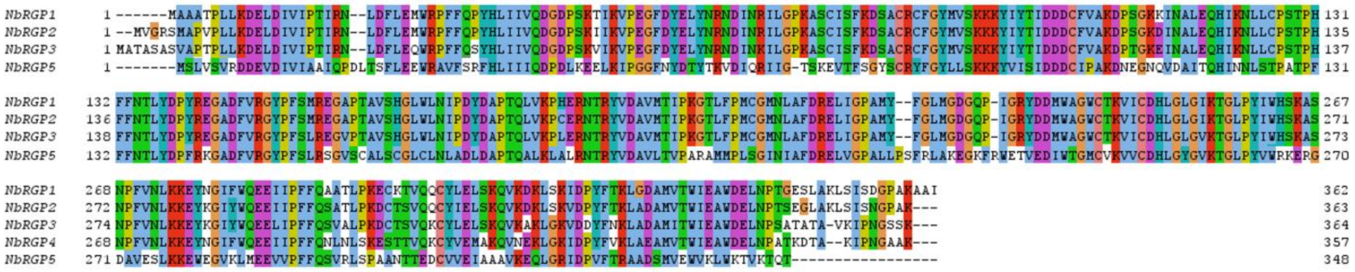

Amino Acid Sequence Identities of NbRGP Proteins

	NbRGP1	NbRGP2	NbRGP3	NbRGP5
NbRGP1	-	94.72%	87.43%	48.80%
NbRGP2	94.72%	-	92.46%	48.87%
NbRGP3	87.43%	92.46%	-	50.30%
NbRGP5	48.80%	48.87%	50.30%	-

Figure S1. Multiple alignment of NbRGP amino acid sequences (top) and the percent of similarity (bottom).

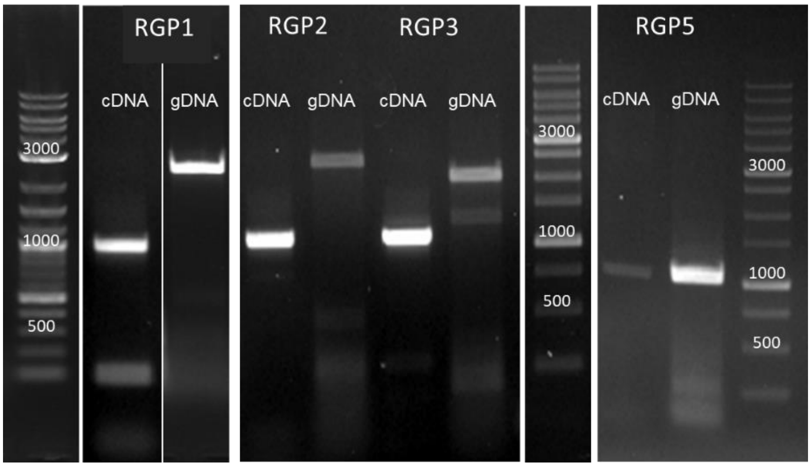

Figure S2. Comparison of PCR products obtained from cDNA and genomic DNA (gDNA) for each NbRGP.

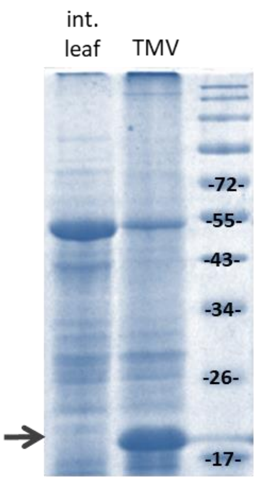

Figure S3. **TMV systemic infection**. Total soluble protein extracted from the intact leaf (int. leaf) and leaf with systemic TMV infection separated in PAAG and stained with Coomassie blue. The arrowhead indicates the band corresponding to TMV coat protein.

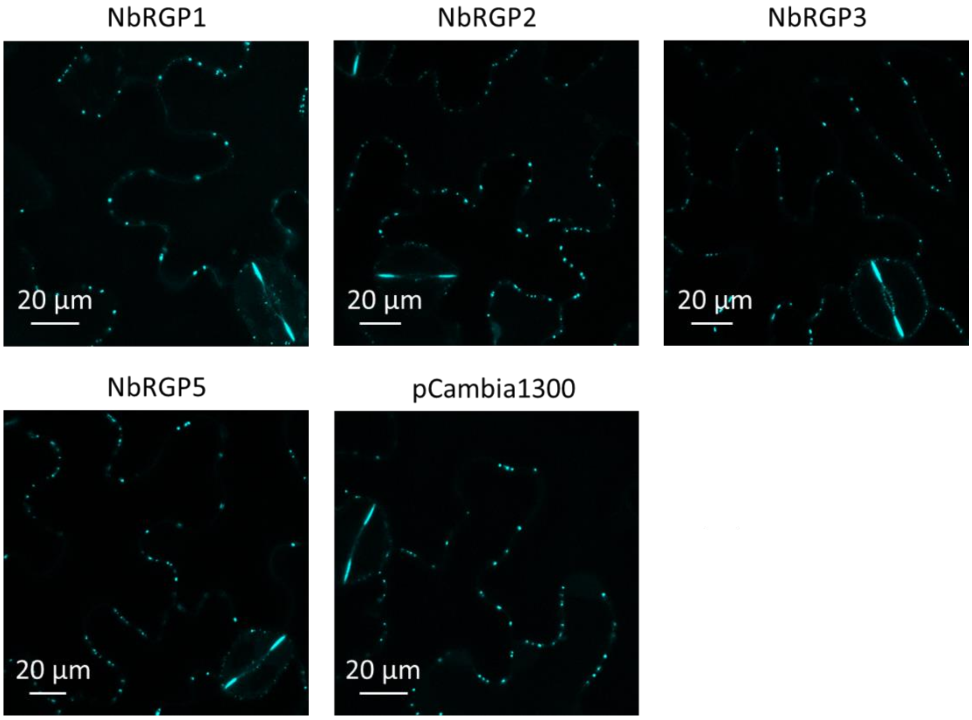

Figure S4. Confocal images of epidermal cells with *NbRGPs* overexpression after callose staining with aniline blue.

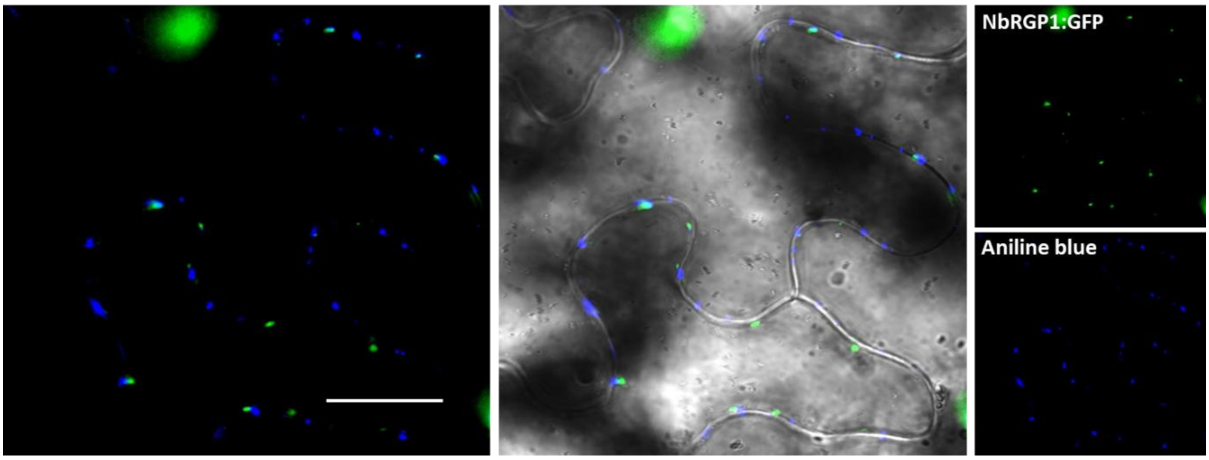

Figure S5. Confocal images of epidermal cells expressing 35S-NbRGP1:GFP (green) and stained with aniline blue (blue).

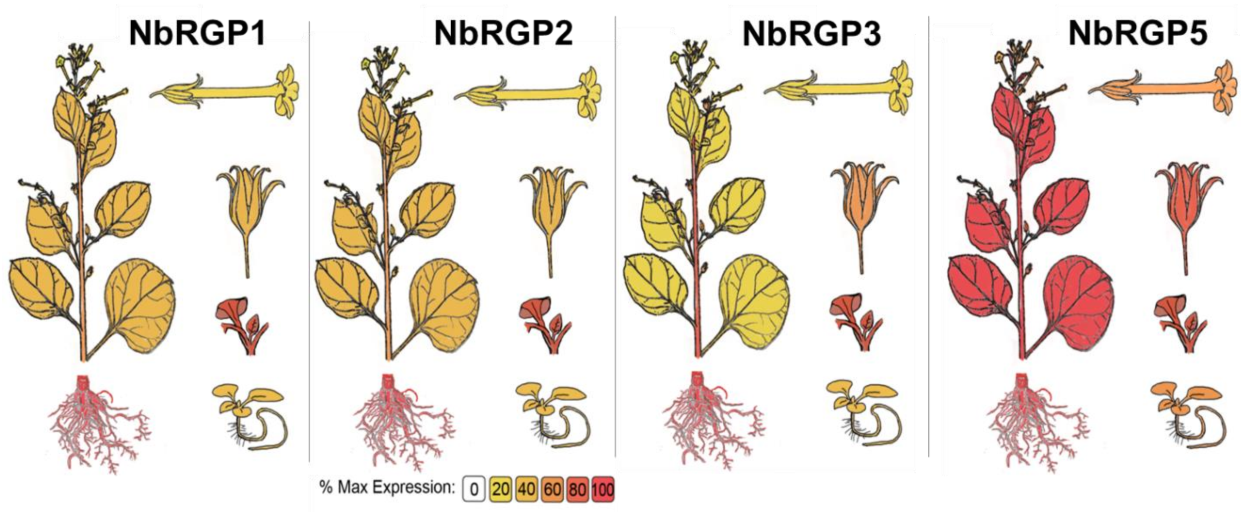

Figure S6. *NbRGPs* patterns of expression according to Version 6 Gene expression Atlas https://sefapps02.qut.edu.au/atlas/tREX6.php)

Table S1. Oligonucleotides used for cloning

F1	GGTACCATGGCAGCAGCAACGCCATTG
F2	GGTACCATGGTAGGACGATCAATG
F3	TCGCGATGGCAACGGCTTC
F4	GGGCCCGTGGTGAGCAAGGGCGAGGAG
F5	GGTACCATGTCGCTAGTCAGTG
F6	GGGCCCGCCGACAAGCAGAAGAACGG
F7	GGTACCATGGCTCTAGTTGTTAAAGG
F8	GGTACCGCAGCAGCAACGCCATTG
F9	GGATCCATAAGAACGGGGCCCAGAGTGAG
F10	GGATCCATAAGAACGGGGCCCATCGCCTCCTC
F11	GGTACCATGTCGTCCTCTCGATTCGATTC
F12	GGATCCATAAGAACGGGGCCCATCGCCTCCTC
F13	GGTACCATGAAGATCATATCAAGGA
R2	GTCGACCTACTTTGCCGGGCCATTTG
R3	GTCGACTATTTTGAGCTGCCATTGG
R4	GGATCCCGACTTTGCAGCCTTTGCAGG
R5	GTCGACTTAAGTTTGGGTTTTGACAG
R9	GTCGACTTACTTGTACAGCTCGTCCA
R10	GTCGACTTAGGCGCCGGTGGAGTG
R11	GTCGACTTACATGATATAGACGTTGTGGCTGTTGTAG
R12	GTCGACTTAGGACTTGTACAGCTCGTCCATGCC
R13	GGATCCAAACGAATCCGATTCGGC
R14	GGATCCTACAACAGAAGCTAGTTTCCCCG
R15	GTCGACTTAGGCGCCGGTGGAGTG
R16	GGATCCAACCTTCTTGAACACAAT

Table S2. Oligonucleotides used for qRT-PCR

Gene	Forward primer	Reverse primer
18S rRNA	ACGGCTACCACATCCAAG	ACTCATTCCAATTACCAGACTC
PP2A	ATTGCTGCCTGTGGTTATTAC	ATAGACTGAAGTGCTTGATTGG
NbRGP1	GGAATGAACTTGGCCTTTGACC	GCCCACATATCATCGTAACGAC
NbRGP2	GATTCTGCTTGTCGGTGCTT	CACGAACGAAATCTGCACCT
NbRGP3	TCGAGGATTCTGCTTGCCGT	TCCTTGCCAGTTGGGTCTTT
NbRGP5	CTGGCGCTGAGGAACACT	ATGGTAGCAAAGCAGGTCCC
MP	GGTGTGAGCGTGTGTCTGG	GCGTCCTGGGTGGTTATAGC
GFP	GCAGAAGAACGGCATCAAG	GCTCAGGTAGTGGTTGTCG
